# The Real-World Outcomes of Relapsed/Refractory Multiple Myeloma Treated with Elotuzumab, Pomalidomide, and Dexamethasone

**DOI:** 10.3390/hematolrep16040058

**Published:** 2024-09-30

**Authors:** Hitomi Nakayama, Yoshinobu Aisa, Chisako Ito, Aki Sakurai, Tomonori Nakazato

**Affiliations:** Department of Hematology, Yokohama Municipal Citizen’s Hospital, 1-1 Mituzawa-Nishicho, Kanagawa-ku, Yokohama-shi 221-0855, Kanagawa, Japan; hnakaymch@gmail.com (H.N.); yoshinobuaisa@aol.com (Y.A.); chisako-ito@i.softbank.jp (C.I.); yamamotoaki1012@gmail.com (A.S.)

**Keywords:** multiple myeloma, elotuzumab, pomalidomide, relapse/refractory, transplant ineligible

## Abstract

**Introduction**: A combination of elotuzumab, pomalidomide, and dexamethasone (EPd) was approved for the treatment of relapsed/refractory multiple myeloma (RRMM) following the ELOQUENT-3 phase II clinical trial. However, the clinical experience with this therapy is still limited. In this retrospective study, we analyzed the efficacy and safety of EPd in a real-world cohort of RRMM patients. **Patients and Methods**: The medical records of 22 patients who received EPd for RRMM at Yokohama Municipal Citizen’s Hospital (Japan) between January 2020 and July 2021 were reviewed. **Results**: The median age of our cohort was 73.5 years. The overall response rate was 55%. With a median follow-up of 20.2 months, the median progression-free survival (PFS) was 9.1 months (95% confidence interval [CI], 2.5–23.0 months). The median PFS was shorter in patients with a poor performance status (PS) than in those with favorable PS (2.5 vs. 10.8 months; *p* < 0.01). Patients with prior daratumumab had significantly shorter PFS than those without prior daratumumab (2.1 vs. 23.0 months; *p* < 0.01). Additionally, patients with prior pomalidomide had significantly shorter PFS (1.7 vs. 10.3 months; *p* < 0.01). In the multivariate analysis, poor PS (hazard ratio [HR] = 4.1, 95% CI: 1.1–15.6; *p* = 0.04) and prior exposure to daratumumab (HR = 3.8, 95% CI: 1.1–13.8; *p* = 0.04) remained significantly associated with shorter PFS. **Conclusions**: The results of our study suggest that EPd is an active and well-tolerated regimen in RRMM, even in real-world patients. Furthermore, EPd may be useful, especially in daratumumab-naïve patients.

## 1. Introduction

Multiple myeloma (MM) is a clonal plasma cell proliferative disorder characterized by clonal expansion of malignant plasma cells in the bone marrow, leading to anemia, lytic bone lesions, hypercalcemia, and elevated M protein in blood or urine, and it is associated with renal dysfunction [1,2]. The treatment outcome of patients with MM has significantly improved in the past two decades with the introduction of novel agents, such as proteasome inhibitors (PIs [e.g., bortezomib, carfilzomib, ixazomib]), immunomodulatory agents (IMIDs [e.g., thalidomide, lenalidomide, pomalidomide]), and monoclonal antibodies (MoAbs [e.g., elotuzumab, daratumumab, isatuximab]) [3,4,5,6,7,8]. However, most patients relapse and eventually become refractory to available anti-MM agents with successive lines of therapy. The long-term outcomes in patients with relapsed/refractory multiple myeloma (RRMM) are still poor [9,10,11]. Moreover, older patients show shorter survival [6,7], as they often present with comorbidities and frailty that may have been associated with the disease and prior lines of therapy. Since the patient-related and disease-related factors of RRMM patients are heterogeneous, the treatment options for individual patients are of growing interest.

Elotuzumab is a humanized monoclonal antibody targeting the signaling lymphocytic activation molecule F7, a glycoprotein that is highly expressed on the surface of myeloma cells, as well as natural killer cells (NK cells) [12,13]. Elotuzumab induces myeloma cell death through multiple mechanisms, including direct NK cell activation, NK cell-mediated antibody-dependent cellular cytotoxicity (ADCC), and macrophage-mediated antibody-dependent cellular phagocytosis [12,13,14,15,16,17]. The combination of elotuzumab and pomalidomide may have synergistic clinical effects in patients with multiple myeloma that relapsed after treatment with lenalidomide or is refractory to lenalidomide. Because immunomodulatory drugs act through several mechanisms, pomalidomide may enhance the immune cell-mediated killing of myeloma cells by elotuzumab. Recently, the efficacy and safety of elotuzumab in combination with pomalidomide and dexamethasone (EPd) in RRMM was reported in the randomized phase II ELOQUENT-3 study [18,19]. In that clinical trial, EPd demonstrated a favorable response with an overall response rate (ORR) of 53% [18]. Moreover, significant improvements in both progression-free survival (PFS) and overall survival (OS) were also observed [18,19].

However, clinical trials have an inherent problem in their strict eligibility criteria, which precludes the inclusion of elderly/frail individuals and individuals with comorbidities, making it difficult to generalize the results. Several studies have shown that a substantial population of patients with MM are ineligible for inclusion in clinical trials and that these patients have worse survival [20,21,22]. Thus, the clinical efficacy and safety of EPd in most RRMM patients are not well described, and real-world data are warranted.

In this retrospective study, we described the real-world use of EPd and evaluated its effectiveness and safety in a real-world cohort of RRMM patients. In addition, we further investigated the factors affecting the outcome of this therapy.

## 2. Patients and Methods

### 2.1. Patients

We retrospectively reviewed the medical records of 22 consecutive patients who received EPd for RRMM at Yokohama Municipal Citizen’s Hospital (Kanagawa, Japan) between January 2020 and July 2021. All patients were diagnosed with symptomatic multiple myeloma according to the IMWG criteria [2] and treated with at least one previous regimen. Patient information was retrieved for analysis on 30 March 2022. Although the number of patients is small (22 cases), this study is considered to be of high clinical significance because there are few data about the efficacy and safety of EPd in real-world clinical practice. This study was approved by the institutional review board of Yokohama Municipal Citizen’s Hospital. Given the retrospective nature of this research study, the ethics committee waived the requirement for formal informed consent, and only de-identified data were evaluated.

### 2.2. Treatment and Dose Adjustment

Patients received 10 mg/kg intravenous elotuzumab on days 1, 8, 15, and 22 during cycles 1 and 2 and 20 mg/kg on day 1 of each cycle thereafter. Pomalidomide was administered orally at a dose of 4 mg/day on days 1–21 of each cycle. Dexamethasone was given both orally (28 mg in patients ≤ 75 years of age; 8 mg in patients > 75 years of age) and intravenously (8 mg) on elotuzumab treatment days and at doses of 40 mg (patients ≤ 75 years of age) or 20 mg (patients > 75 years of age) weekly on days without elotuzumab. Pomalidomide and dexamethasone were reduced from the starting dose according to age and frailty at the discretion of each physician [23]. All patients received acetylsalicylic acid for prophylaxis against thromboembolism. Treatment was continued until disease progression or unacceptable toxicity. Responses were defined according to the IMWG criteria [24]. Adverse events (AEs) were recorded and graded according to the National Cancer Institute Common Terminology Criteria for Adverse Events, v. 4.0.

### 2.3. Statistical Analysis

OS was calculated from the start of the treatment until the date of death or the date the patient was last known to be alive. PFS was calculated from the start of the treatment until the date of disease progression or death (regardless of the cause of death). The probability of PFS and OS was estimated according to the Kaplan–Meier method and compared among groups using the log-rank test. Univariate and multivariate analyses for factors affecting PFS were performed using a Cox proportional hazards model. Variables with *p* values of ≤0.01 in the univariate analysis were included in the multivariate model. All statistical analyses were performed with EZR (Saitama Medical Center, Jichi Medical University, Saitama, Japan) [25]. *p* values of <0.05 were considered to indicate statistical significance.

## 3. Results

### 3.1. Patient Characteristics and Treatment Exposure

The characteristics of the 22 evaluated patients are summarized in Table 1. The median age of the patients was 73.5 years. Half of the patients (50%) were classified as International Staging System (ISS) stage III. The median number of prior treatment lines was four (range 1–10). All but one (96%) patient had been exposed to bortezomib, and twelve (55%) had been exposed to carfilzomib or ixazomib. Eighteen (82%) patients had been exposed to lenalidomide, six of whom had also been exposed to pomalidomide during any previous regimen. Ten (46%) patients had previously undergone autologous stem cell transplantation, three (14%) of whom also received allogeneic hematopoietic stem cell transplantation. Nine (41%) patients had received prior daratumumab, and only one patient had been exposed to isatuximab. Four (18%) patients had received prior radiotherapy.

At the time of the database lock, treatment was discontinued in 15 (78%) of the 22 treated patients. The most common reason for treatment discontinuation was disease progression, which occurred in thirteen (59%) patients, a decrease in the performance status (PS) in one patient, and a request to stop treatment in one patient. The median number of treatment cycles was five (range 1–25).

The median relative dose intensity (RDI) of elotuzumab was 60% (range 17–98%); 36% of patients received an RDI of ≥80%. Seven of the twenty-two patients (32%) had received monthly dosing of elotuzumab from the second cycle (20 mg/kg of elotuzumab on day 1 of each cycle) due to the difficulty in frequent hospital attendance. The starting dose of pomalidomide was reduced in 17 (77%) of the 22 treated patients: 11 at 2 mg/day and the other 6 at 3 mg/day; the median RDI of pomalidomide was 42% (range 6–89%). The median RDI of dexamethasone in patients of ≤75 years of age was 42% (range 5–69%), while that in patients of >75 years of age was 40% (range 9–88%).

### 3.2. Efficacy

Twelve of the twenty-two treated patients achieved partial response or better (ORR: 55%). Six patients had stable disease, and four had progressive disease.

**Figure 1:**
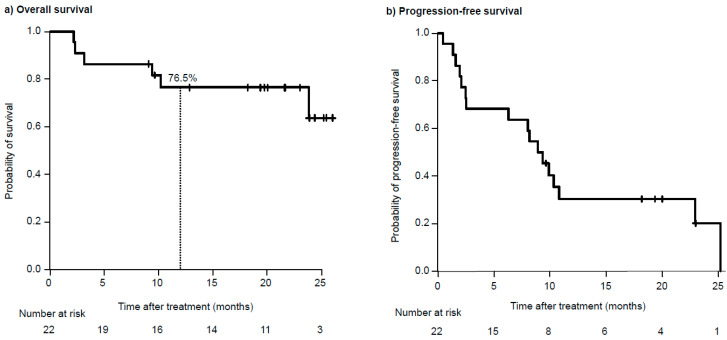
Kaplan–Meier curves for (**a**) overall survival and (**b**) progression-free survival of the whole cohort of patients treated with elotuzumab, pomalidomide, and dexamethazone.

**Figure 2:**
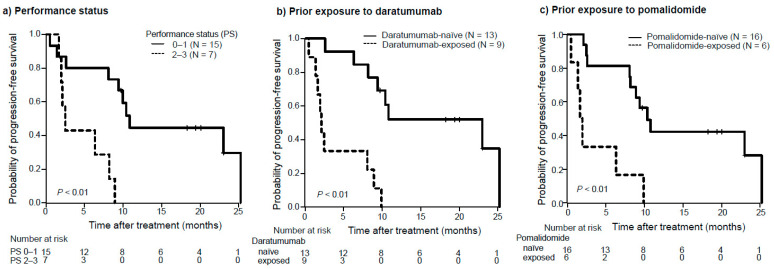
Progression-free survival with elotuzumab, pomalidomide, and dexamethazone among the patient groups. Progression-free survival (PFS) was compared between the patient groups. (**a**) Eastern Cooperative Oncology Group (ECOG) performance status (PS) 0–1 vs. PS 2–3. (**b**) Daratumumab-naïve vs. daratumumab-exposed. (**c**) Pomalidomide-naïve vs. pomalidomide-exposed.

With a median follow-up of 20.2 months (range 2.2–26.4), six patients (27%) died, all due to disease progression. The median OS (95% confidence interval [CI]) was not reached (23.8 months; not estimable), and the OS rate at 1 year was 76.5% (Figure 1a). The median PFS was 9.1 months (95% CI, 2.5–23.0 months) (Figure 1b). The median PFS was shorter in patients with Eastern Cooperative Oncology Group (ECOG) PS 2–3 than in those with PS 0–1 (2.5 vs. 10.8 months; *p* < 0.01) (Figure 2a). Patients who had received prior daratumumab had significantly shorter PFS than those who had not (2.1 vs. 23.0 months; *p* < 0.01) (Figure 2b). Additionally, patients with prior pomalidomide had significantly shorter PFS than those without (1.7 vs. 10.3 months; *p* < 0.01) (Figure 2c). Other factors, including sex, ISS stage, number of prior therapies, prior exposure to lenalidomide, second-generation PIs (carfilzomib and ixazomib), and history of autologous stem cell transplantation, had no significant association with PFS. As shown in Table 2, the multivariate analysis demonstrated that higher ECOG PS (hazard ratio [HR] = 4.1, 95% CI: 1.1–15.6; *p* = 0.04) and prior exposure to daratumumab (HR = 3.8, 95% CI: 1.1–13.8; *p* = 0.04) remained significantly associated with shorter PFS. 

### 3.3. Safety

The adverse events (AEs) in our cohort are shown in Table 3. The most common any-grade AEs were anemia (91%) and lymphocytopenia (91%). The most common grade 3/4 AEs were lymphocytopenia (64%) and neutropenia (59%). Infections occurred in 10 of the 22 (45%) treated patients, 7 (32%) of which were grade 3/4. Three (14%) patients had cytomegalovirus reactivation. Five (23%) patients experienced infusion-related reactions, all of which were grade 1/2 and occurred during the first treatment cycle. AEs leading to treatment interruption occurred in eight (36%) patients. The most common reason for treatment interruption was infection, which occurred in five (23%) patients, sick sinus syndrome in one patient, pneumonitis in one patient, and skin rash in one patient. In all patients, the resolution of symptoms was achieved with appropriate treatment. There were no treatment-related deaths in our cohort.

## 4. Discussion

Recent reports of the phase II randomized clinical trial of elotuzumab in combination with pomalidomide/dexamethasone (Pd) demonstrated a significant improvement in both PFS and OS in comparison to Pd in patients with RRMM [18,19]. In this retrospective observational study, we described our real-world experience with EPd for the treatment of RRMM. Our results also showed that EPd is used effectively and safely in clinical practice in patients with RRMM, with notable differences from the clinical trial population.

In comparison to the published dataset of the ELOQUENT-3 study [18,19], the 22 patients of our real-world cohort were older (median, 73.5 vs. 68.5 years) and included patients with ECOG PS scores of 3. Furthermore, our study showed a higher proportion of patients classified as ISS stage III (50% vs. 12%), and the patients were more heavily pretreated (median, 4 vs. 3 prior lines). Remarkably, 41% (N = 9) of them had been exposed to daratumumab, and 27% (N = 6) of them had previously received pomalidomide. These results of our study were consistent with previous studies reporting that, in clinical practice, patients often have a high tumor burden and are not as healthy as clinical trial populations [20,21,22]. Even so, the responses and efficacy observed in our results were largely similar to those observed in the ELOQUENT-3 study, with an ORR of 55% in our study and 53% in the ELOQUENT-3 study [18]. Despite the short observation period, the survival curve was almost identical to that of the ELOQUENT-3 study. The median PFS in our study was comparable to that observed in the ELOQUENT-3 study (9.1 vs. 10.3 months) [18]. In our cohort, the OS rate at 1 year was also similar or slightly lower than that in the ELOQUENT-3 study (76.5% vs. 79%) [19]. The results of our study, which suggest that EPd provides both PFS and OS benefits in a real-world RRMM patient population, are of great clinical significance.

The responses and efficacy observed in our findings were more favorable than those observed in a prior real-world study. A recent retrospective analysis of 22 patients who received EPd in Germany and Austria (two-thirds of whom had previously received pomalidomide) reported an ORR of 50% and a median PFS of 6.4 months [26]. While the ORR was comparable to that of ELOQUENT-3 and our study, the lower PFS of that study may reflect the fact that patients in that study were more heavily pretreated, with a median of five prior lines, including pomalidomide-containing therapy. Another recent multicenter retrospective analysis of 200 patients who received EPd in Italy reported an ORR of 55.4% and a median PFS of 7 months [27]. The PFS of the study was also inferior to that of ELOQUENT-3 because 73% of the patients had previously received daratumumab. 

Regarding the safety profile, the most common AEs were anemia and lymphocytopenia, which occurred in 91% of the treated patients. In comparison to the ELOQUENT-3 study [19], all hematological AEs were more frequently detected in our cohort: any grade anemia (28% vs. 91%), neutropenia (27% vs. 77%), thrombocytopenia (17% vs. 55%), and lymphocytopenia (10% vs. 91%). These results may reflect the fact that the patients in our study were older and more heavily pretreated than the patients in ELOQUENT-3. Despite the high incidence of neutropenia and lymphocytopenia, a smaller proportion of patients in our study developed infections (any grade 45% vs. 70%), while the rate of grade 3/4 infections was comparable to that reported in the ELOQUENT-3 study (32% vs. 25%) [19]. The results of our study indicate that EPd is an active and well-tolerated regimen in real-world RRMM patients.

Because of the paucity of published studies on real-world RRMM patients treated with EPd, we performed a subgroup analysis of this heavily pretreated and very frail patient population. In our cohort, a multivariate analysis identified that a higher ECOG PS score (≥2) and prior exposure to daratumumab were significant factors associated with shorter PFS. Given the poor outcomes of these very frail patients, treatment indications for these patients should be carefully considered. However, while patients with ECOG PS > 2 were excluded from the ELOQUENT-3 study [18,19], one-third of our real-world patients had ECOG PS ≥ 2, indicating the limited generalizability of the results of the randomized clinical trial.

Our results also showed that the patients who had received prior daratumumab had significantly shorter PFS than those who had not (HR = 3.8; 95% CI, 1.1–13.8; *p* = 0.04). However, prior exposure to lenalidomide or second-generation PIs had no significant association with PFS. The results of our study suggest that EPd is an effective therapy even in patients who are refractory to lenalidomide and second-generation PIs. It was also suggested that EPd may be more effective in patients who are naïve to daratumumab. A possible mechanism for the association between daratumumab exposure and EPd outcomes could be that the depletion of NK cells by daratumumab [28,29] resulted in impaired NK cell activity and NK cell-mediated ADCC, which are the original effects of elotuzumab [12,13,14,15,16]. This result in our study is consistent with that of the MAMMOTH study, a retrospective study of patients with MM refractory to anti-CD38 MoAbs that showed limited efficacy of elotuzumab-based treatment in anti-CD38 MoAb-refractory MM patients [11]. A recent Italian multicenter retrospective study of 321 RRMM patients who received at least one cycle of EPd reported an ORR of 55.4% and a median PFS of 7.5 months. Notably, nearly 77% of cases had been exposed to daratumumab [30]. All 247 daratumumab-exposed patients were refractory to daratumumab. This study demonstrated that advanced ISS II/III stage and previous exposure to daratumumab were confirmed as independent prognostic factors on PFS in the multivariate analysis. They speculated that the depletion of CD38+ NK cells by daratumumab exposure is associated with the low efficacy of EPd.

Daratumumab is now being used in a substantial population of patients with the recent approval of its use in both transplant-ineligible newly diagnosed MM patients [31,32] and RRMM patients [33]. Although daratumumab is now represented as standard, first-line therapy for transplant-ineligible MM patients [31,32], a considerable population of real-world patients receive two-drug regimens as first-line therapy (e.g., bortezomib-dexamethasone or lenalidomide-dexamethasone) due to their age and frailty [8,34]. The results of our study suggest that EPd may be an attractive treatment option in this patient group after they become refractory to their first-line doublet therapy.

The results of our study provide important information regarding the real-world use of elotuzumab and pomalidomide. The median RDI of elotuzumab in our study was 60% (range 17–98%). The decrease in the RDI of elotuzumab was caused by the delay in the treatment interval: a noticeably high proportion (32%) had monthly dosing of elotuzumab from the second cycle. Thus, only 36% of patients achieved an RDI of ≥80%. The starting dose of pomalidomide was also reduced in 77% of the patients in our study, with a median RDI of 42%, which was lower than that reported in the ELOQUENT-3 study (51.7%) [19]. This observed dosing schedule reduction may have been for convenience or to reduce the treatment burden on these elderly and frail patients. Although most of our patients received dose modifications based on a clinician’s judgment, our results suggest that the overall efficacy and safety may not be compromised despite these changes.

The present study was associated with several limitations, some of which arise from its retrospective nature. Because of missing data, we failed to assess the impact of high-risk disease features, such as high-risk cytogenetics, on the outcome. We also failed to capture some variables, particularly regarding frail elderly patients, which are not uniformly collected but which could potentially influence the outcome in a real-world setting. Only the PS score could be evaluated in our study; however, information from a comprehensive geriatric assessment may have provided more detailed patient profiles and their outcomes [35]. Another limitation was the relatively small study population, which may have influenced the findings, particularly those related to outcomes from Cox models. Thus, the findings from the subgroup analysis of PFS are limited and should be interpreted with caution.

## 5. Conclusions

Our results demonstrated that EPd is effective and has an acceptable safety profile in real-world RRMM patients, including those who are elderly, frail, lenalidomide-refractory, and have a high tumor burden. Moreover, patients who were exposed to daratumumab or pomalidomide were also included in our study. Despite the increasing number of new therapeutic strategies for RRMM, this study may provide clinicians with better insight regarding the use of EPd in the relapsed/refractory setting. A future prospective study with larger patient groups is warranted to better understand patient and disease predictors of this therapy and to elucidate strategies for optimizing elotuzumab use in the real world. 

## Figures and Tables

**Table 1:** Patient characteristics (N = 22).

Median Age, Years (Range)	73.5	(50–84)
Male sex, no (%)	6	(27)
ECOG performance status, no (%)		
0	12	(55)
1	3	(14)
2	4	(18)
3	3	(14)
Type of myeloma, no (%)		
IgG	10	(46)
IgA	6	(27)
Light chain	6	(27)
**International Staging System (ISS) stage**, no (%)		
I or II	11	(50)
III	11	(50)
**Cytogenetic abnormalities (Del17p, t(4;14), or t(14;16))**, no (%)		
Yes	3	(14)
No	7	(32)
Data not available	12	(55)
**Extramedullary disease**, no (%)		
Yes	3	(14)
No	19	(86)
**Amyloidosis**, no (%)		
Yes	3	(14)
No	19	(86)
Central nervous system involvement, no (%)		
Yes	0	(0)
No	22	(22)
Comorbidities, no (%)		
Heart failure	3	(14)
Chronic obstructive pulmonary disease	2	(9)
Other malignancy	2	(9)
Median no. of previous lines of therapy, (range)	4	(1–10)
Previous therapies, no (%)		
Bortezomib	21	(96)
Lenalidomide	18	(82)
Daratumumab	9	(41)
Ixazomib	9	(41)
Pomalidomide	6	(27)
Carfilzomib	3	(14)
Isatuximab	1	(5)
Autologous stem cell transplantation	10	(46)
Allogeneic stem cell transplantation	3	(14)
Radiotherapy	4	(18)
Median time since initial diagnosis, months (range)	52.6	(4.7–129.6)

ECOG, Eastern Cooperative Oncology Group.

**Table 2:** Univariate and multivariate analyses of the association between various factors and progression-free survival.

	Univariate			Multivariate		
Factors	Hazard Ratio	95% CI	*p* Value	Hazard Ratio	95% CI	*p* Value
**ECOG performance status**	6.3	1.8–22.4	<0.01	4.1	1.1–15.6	0.04
0–1 versus 2–3						
**Prior treatment with daratumumab**						
No versus Yes	8.3	2.5–28.2	<0.01	3.8	1.1–13.8	0.04
**Prior treatment with pomalidomide**						
No versus Yes	5.8	1.9–17.9	<0.01			

CI, confidence interval; ECOG, Eastern Cooperative Oncology Group.

**Table 3:** Adverse events (N = 22).

Events	Any Grade		Grade 3 or 4	
	No. of Patients (%)			
**Hematological adverse events**				
Anemia	20	(91)	5	(23)
Neutropenia	17	(77)	13	(59)
Lymphocytopenia	20	(91)	14	(64)
Thrombocytopenia	12	(55)	4	(18)
**Nonhematological adverse events**				
Infection	10	(45)	7	(32)
Infusion reaction	5	(23)	0	(0)
Peripheral neuropathy	5	(23)	0	(0)
Skin rash	3	(14)	0	(0)

## Data Availability

The data supporting the findings of this study are available from the corresponding author upon reasonable request.
